# Association between miR-124-1 rs531564 polymorphism and risk of cancer: An updated meta-analysis of case-control studies

**DOI:** 10.17179/excli2018-1419

**Published:** 2018-06-28

**Authors:** Abdolkarim Moazeni-Roodi, Mohammad Hashemi

**Affiliations:** 1 Department of Clinical Biochemistry, Iranshahr University of Medical Sciences, Iranshahr, Iran; 2 Department of Clinical Biochemistry, School of Medicine, Zahedan University of Medical Sciences, Zahedan, Iran

**Keywords:** miR-124-1, rs531564, polymorphism, cancer, meta-analysis

## Abstract

Many studies examined the association between miR-124-1 rs531564 polymorphism and the risk of some human cancers, but the findings remain controversial. This update meta-analysis aimed to validate the association between rs531564 polymorphism of miR-124-1 and cancer risk. Eligible studies including 6,502 cancer cases and 7,213 controls were documented by searching Web of Science, PubMed, Scopus, and Google scholar databases. Pooled odds ratios (ORs) with 95 % confidence intervals (CIs) were estimated to quantitatively evaluate the association between rs531564 variant and cancer risk. The results indicated that rs531564 variant significantly decreased the risk of cancer in homozygous codominant (OR=0.54, 95 % CI=0.43-0.69, p<0.00001, GG vs CC), dominant (OR=0.84, 95 % CI=0.72-0.99, p=0.03, CG+GG vs CC), recessive (OR=0.65, 95 % CI=0.54-0.78, p<0.00001, GG vs CG+CC), and allele (OR=0.84, 95 % CI=0.73-0.96, p=0.008, G vs C) genetic model. Stratified analysis by cancer type revealed that rs531564 variant was associated with gastric cancer, cervical cancer, esophageal squamous cell carcinoma and colorectal cancer risk. In summary, the findings of this meta-analysis support an association between miR-124-1 rs531564 polymorphism and cancer risk. Larger and well-designed studies are required to estimate this association in detail.

## Introduction

Cancer is one of the main leading cause of morbidity and mortality worldwide (Global Burden of Disease Cancer Collaboration, 2015[11]). In 2016, approximately 17.2 million new cancer cases and 8.9 million deaths occurred worldwide (Global Burden of Disease Cancer Collaboration, 2018[10]). It has been proposed that the complex interaction of various genetic loci and diverse environmental factors play a role in cancer development (Borek, 1993[2]; Lichtenstein et al., 2000[18]). Despite physical disparities, all human populations are 99 % genetically identical, and the remaining 1 % genetic variations is responsible for human diversity (International HapMap Consortium, 2007[14]; Ryan et al., 2010[25]). Single-nucleotide polymorphisms (SNPs) contribute to phenotypic differences both within and among populations (Omrani et al., 2014[23]). 

MicroRNA (miRNA) are a class of noncoding RNAs consisting of 18-25 nucleotides in length that bind to the 3`untranslated region (3'UTR) of target mRNAs to regulate gene expression (Ryan et al., 2010[25]). Variations in miRNAs genes, including pri-miRNAs, pre-miRNAs, and mature miRNAs, impact on miRNAs biogenesis, processing, target binding, and expression level of mature miRNAs (Mishra et al., 2008[22]). Preceding studies have shown that miRNAs play a crucial role in various tumor-associated biological processes, including proliferation, metastasis, apoptosis and differentiation (He et al., 2013[12]; Liu et al., 2013[19]; Ge et al., 2016[9]). 

In human, miR-124 is encoded by three miRNA genes including MIR124-1 (8p23.1), MIR124-2 (8q12.3), and MIR124-3 (20q13.33). A functional polymorphism rs531564 located in the pri-miRNA region of the miR124-1 gene affect the expression levels of the mature miR-124 (Qi et al., 2012[24]). To date, several epidemiological studies inspected the association between miR-124-1 rs531564 polymorphism and the risk of various cancer including gastric cancer (Zhou et al., 2012[37]; Asgarpour et al., 2017[1]; Singh et al., 2017[28]), cervical cancer (Wu and Zhang, 2014[31]; Xiong et al., 2014[32]; Chuanyin et al., 2017[5]), breast cancer (Ma et al., 2013[20]; Ying et al., 2016[34]; Danesh et al., 2018[6]), renal cell carcinoma (Liang et al., 2017[17]), osteosarcoma (Shi et al., 2016[27]), esophageal squamous cell carcinoma (ESCC) (Yin et al., 2013[33]; Zhang et al., 2014[36]; Wu et al., 2018[31]), colorectal cancer (Gao et al., 2015[8]), but the findings are still controversial. Therefore, we performed an updated meta-analysis to find out the impact of rs11134527 polymorphism on cancer risk.

## Methods

### Literature search

A comprehensive search in Web of Science, PubMed, Scopus, and Google Scholar databases was conducted for all articles describing an association between miR-124-1 rs531564 polymorphism and cancer risk published up to June 08, 2018. The search strategy was “cancer OR carcinoma, tumor OR neoplasms”, AND “miR-124-1 OR microRNA-124-1 OR miRNA-124-1” AND “polymorphism OR mutation OR variant OR rs531564”. Relevant studies included the meta-analysis if they met the following inclusion criteria: 1) Original case-control studies that evaluated the miR-124-1 polymorphism and cancer risk; 2) studies provided necessary information of the genotype frequencies of miR-124-1 rs531564 variant in both cases and controls. The exclusion criteria were: 1) conference abstract, case reports, reviews, duplication data; 2) insufficient genotype information provided.

### Data extraction

The authors independently searched the literatures, extracted the relevant data and finally discussed disagreement. The following data were recorded from each study including the first author's name, publication year, country, ethnicity of participants, cancer type, genotyping methods of miR-124-1 rs531564 polymorphism, number of genotypes in case-control groups and result of the HWE test (Table 1(Tab. 1), References in Table 1: Asgarpour et al., 2017[1]; Chuanyin et al., 2017[5]; Danesh et al., 2018[6]; Gao et al., 2015[8]; Liang et al., 2017[17]; Ma et al., 2013[20]; Shi et al., 2016[27]; Singh et al., 2017[28]; Wu et al., 2014[31]; Wu et al., 2018[30]; Xiong et al., 2014[32]; Yin et al., 2013[33]; Ying et al., 2016[34]; Zhang et al., 2014[36]; Zhou et al., 2012[37]).

### Statistical analysis

Meta-analysis was achieved by Revman 5.3 software (Version 5.3. Copenhagen: The Nordic Cochrane Centre, The Cochrane Collaboration, 2014) and STATA 14.1 software (Stata Corporation, College Station, TX, USA). Hardy-Weinberg equilibrium (HWE) for each study was calculated by the χ2 test. The association between miR-124-1 rs531564 polymorphism and cancer risk was assessed by pooled odds ratios (ORs) and their 95 % confidence intervals (CIs) for codominant (CG vs CC and GG vs CC), dominant (CG+GG vs CC), recessive (GG vs CG+CC), overdominant (CG vs CC+GG) and the allelic (G vs C) genetic inheritance models. The significance of the pooled OR was assessed by the Z-test, and P<0.05 was considered to be statistically significant. The choice of using fixed or random effects model was determined by the results of the between-study heterogeneity test, which was measured using the Q test and I^2^ statistic. If the test result was I^2^ ≥ 50 % or P_Q_ < 0.1, indicating the presence of heterogeneity, the random effect model was selected; otherwise, the fixed-effects model was used.

Begg's funnel plot was conducted under all inheritance models to evaluate the publication bias and the asymmetric plots implied potential publication bias. The degree of asymmetry was tested using Egger's test and p < 0.05 was considered significant publication bias. 

Sensitivity analysis was performed to evaluate the stability of the studies on the pooled ORs. A single study in the analysis was neglected each time to calculate the outcomes again.

## Results

### Study characteristics

Totally 15 case-control studies including 6,502 cancer cases and 7,213 controls were included in the meta-analyses. Table 1(Tab. 1) shows the characteristics and relevant data of the included studies.

### Main analysis results

**Figure 1:**
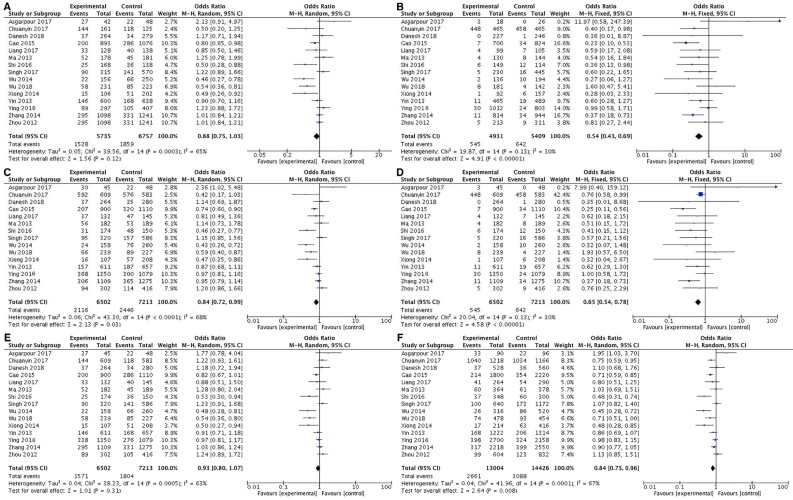
The forest plot for relationship between miR-124-1 rs531564 polymorphism and cancer susceptibility for CG vs CC (A), GG vs CC (B), CG+GG vs CC (C), GG vs CG+CC (D), CG vs CC+GG (E), and G vs C (F)

In the current meta-analysis of 15 eligible studies, the findings support an association between miR-124-1 rs531564 polymorphism and cancer risk. The rs531564 variant significantly decreased the risk of cancer in homozygous codominant (OR=0.54, 95 % CI=0.43-0.69, p<0.00001, GG vs CC), dominant (OR=0.84, 95 % CI=0.72-0.99, p=0.03, CG+GG vs CC), recessive (OR=0.65, 95 % CI=0.54-0.78, p<0.00001, GG vs CG+CC), and allele (OR=0.84, 95 % CI=0.73-0.96, p=0.008, G vs C) inheritance model (Figure 1(Fig. 1) and Table 2(Tab. 2); References in Figure 1: Asgarpour et al., 2017[1]; Chuanyin et al., 2017[5]; Danesh et al., 2018[6]; Gao et al., 2015[8]; Liang et al., 2017[17]; Ma et al., 2013[20]; Shi et al., 2016[27]; Singh et al., 2017[28]; Wu et al., 2014[31]; Wu et al., 2018[30]; Xiong et al., 2014[32]; Yin et al., 2013[33]; Ying et al., 2016[34]; Zhang et al., 2014[36]; Zhou et al., 2012[37]). 

### Subgroup analysis by cancer type

Stratified analysis of miR-124-1 rs531564 polymorphism was achieved by cancer type (Table 3(Tab. 3)). The data implied that rs531564 variant increased the risk of gastric cancer in overdominant (OR=1.27, 95 % CI=1.02-1.58, p=0.03, CG vs GG+CC) inheritance model. The rs531564 variant was associated with significantly decrease in risk of cervical cancer in codominant, dominant, recessive and allele inheritance model (Table 3(Tab. 3)). Furthermore, the variant significantly decreased the risk of ESCC in recessive and allele models. The rs531564 variant was significantly associated with protection against colorectal in recessive model (Table 3(Tab. 3)). No significant association was found between rs531564 variant and breast cancer risk.

### Heterogeneity and publication bias

Heterogeneity among the findings included in the meta-analysis is shown in Table 2(Tab. 2). The data showed heterogeneity existed between studies.

**Figure 2:**
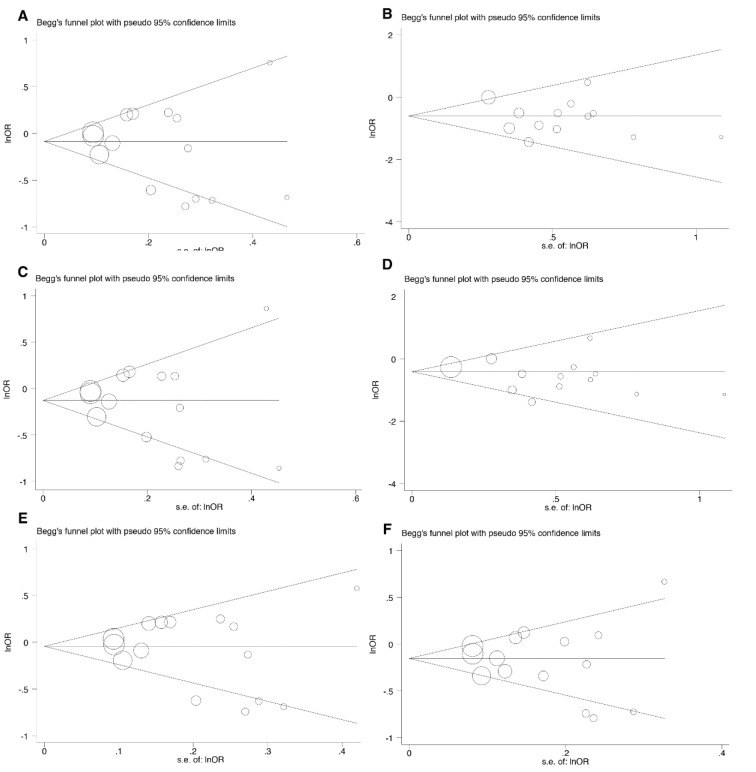
The funnel plot for the test of publication bias. The funnel plot for CG vs CC (A), GG vs CC (B), CG+GG vs CC (C), GG vs CG+CC (D), CG vs CC+GG (E), and G vs C (F)

The potential publication bias was estimated using a Begg's funnel plot and Egger's test. Neither Begg's funnel plot nor Egger's test detected any obvious evidence of publication bias in analyses for all genetic models (Table 2(Tab. 2) and Figure 2(Fig. 2)). 

### Sensitivity analysis

**Figure 3:**
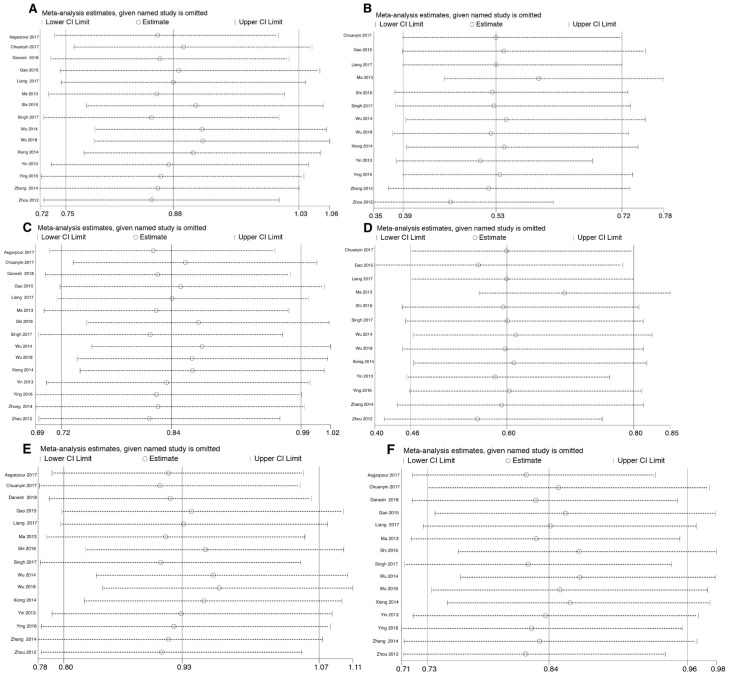
Sensitivity analyses for studies on miR-124-1 rs531564 polymorphism using different genetic models. Sensitivity analyses for CG vs CC (A), GG vs CC (B), CG+GG vs CC (C), GG vs CG+CC (D), CG vs CC+GG (E), and G vs C (F)

We executed sensitivity analysis to evaluate the effect of a specific study on the overall estimate. The relevant pooled ORs showed no significant change appeared when each study was ignored, one at a time, from the overall meta-analysis in homozygous codominant, dominant, recessive, and allele models (Figure 3(Fig. 3), References in Figure 3: Asgarpour et al., 2017[1]; Chuanyin et al., 2017[5]; Danesh et al., 2018[6]; Gao et al., 2015[8]; Liang et al., 2017[17]; Ma et al., 2013[20]; Shi et al., 2016[27]; Singh et al., 2017[28]; Wu et al., 2014[31]; Wu et al., 2018[30]; Xiong et al., 2014[32]; Yin et al., 2013[33]; Ying et al., 2016[34]; Zhang et al., 2014[36]; Zhou et al., 2012[37]). This indicates that the results of this meta-analysis are relatively stable and reliable.

## Discussion

It has been well known that miRNAs is involved in carcinogenesis as tumor suppressor gene or oncogene (Calin et al., 2004[4]; Ryan et al., 2010[25]). Dysregulation of miRNAs contributes to the initiation and progression of human malignancies (Shen et al., 2015[26]; He et al., 2018[13]; Skjefstad et al., 2018[29]). It has been documented that miR-124 is a tumor suppressor miRNA in many cancers (Jin et al., 2017[15]; Yuan et al., 2017[35]; Cai et al., 2018[3]; Ma et al., 2018[21]). 

An increasing number of studies have focused on associations between miR-124-1 rs531564 polymorphism and various cancer susceptibility (Zhou et al., 2012[37]; Ma et al., 2013[20]; Yin et al., 2013[33]; Wu and Zhang, 2014[31]; Xiong et al., 2014[32]; Zhang et al., 2014[36]; Gao et al., 2015[8]; Shi et al., 2016[27]; Ying et al., 2016[34]; Asgarpour et al., 2017[1]; Chuanyin et al., 2017[5]; Liang et al., 2017[17]; Singh et al., 2017[28]; Danesh et al., 2018[6]; Wu et al., 2018[30]), but the findings were inconsistent. We performed an updated meta-analysis of 15 case-control studies to find out the impact of rs531564 polymorphism of miR-124-1 gene on overall cancer risk. 

The findings of our meta-analysis showed that the rs531564 polymorphism was significantly associated with protection against cancer in homozygous codominant, dominant, recessive and allele inheritance model. Our findings are in agreement with the results of two meta-analyses regarding the impact of miR-124-1 rs531564 variant on cancer risk (Fang et al., 2015[7]; Li et al., 2015[16]). The meta-analysis performed by Li et al. (2015[16]) enrolled 4 case-control studies and the findings revealed that rs531564 polymorphism significantly reduced cancer risk. The other study conducted by Fang et al. (2015[7]) with 5 case-control studies also showed that the pri-miR1241 rs531564 polymorphism significantly reduced cancer risk. 

We performed stratified analysis by cancer type and the findings revealed that the rs531564 variant was significantly associated with gastric cancer, cervical cancer ESCC, colorectal and breast cancer risk. 

There are several limitations in our meta-analysis that should be addressed. First, heterogeneity existed between some studies. It can be supposed that the heterogeneity probably derived from difference of ethnicity, source of control, status and cancer type. Second, the languages of the studies were limited to English. Third, we did not evaluate potential gene-environmental interactions due to lack of relevant data across the included studies. Finally, all subjects are of Asian descent, so our meta-analysis was limited to Asian population and lack of other ethnicities. 

In conclusion, our meta-analysis proposed that miR-124-1 rs531564 polymorphism may be an important protective factor for cancer in Asians. Additional well designed case-control studies with larger sample sizes are required to validate the findings.

## Conflict of interest

The authors have declared that no competing interests exist.

## Figures and Tables

**Table 1:**
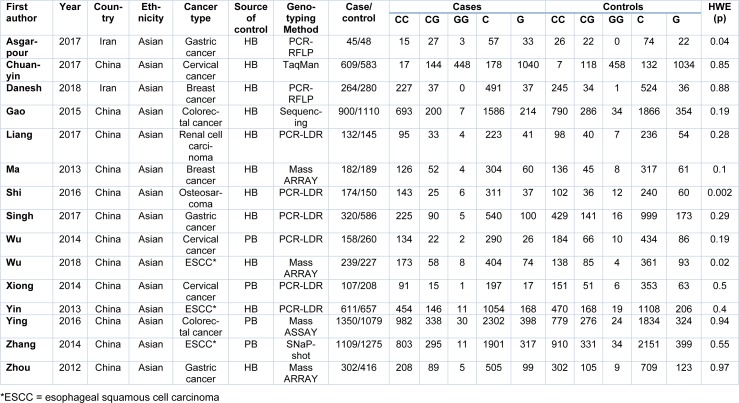
Characteristics of all studies included in the meta-analysis

**Table 2:**
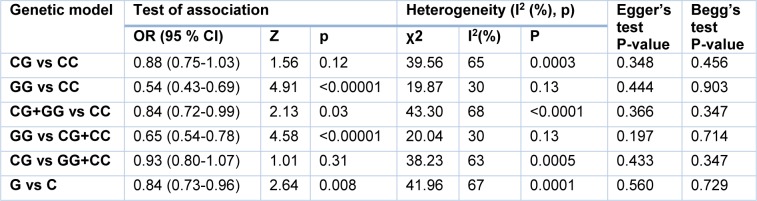
The pooled ORs and 95 % CIs for the association between miR-124-1 rs531564 polymorphism and cancer susceptibility.

**Table 3:**
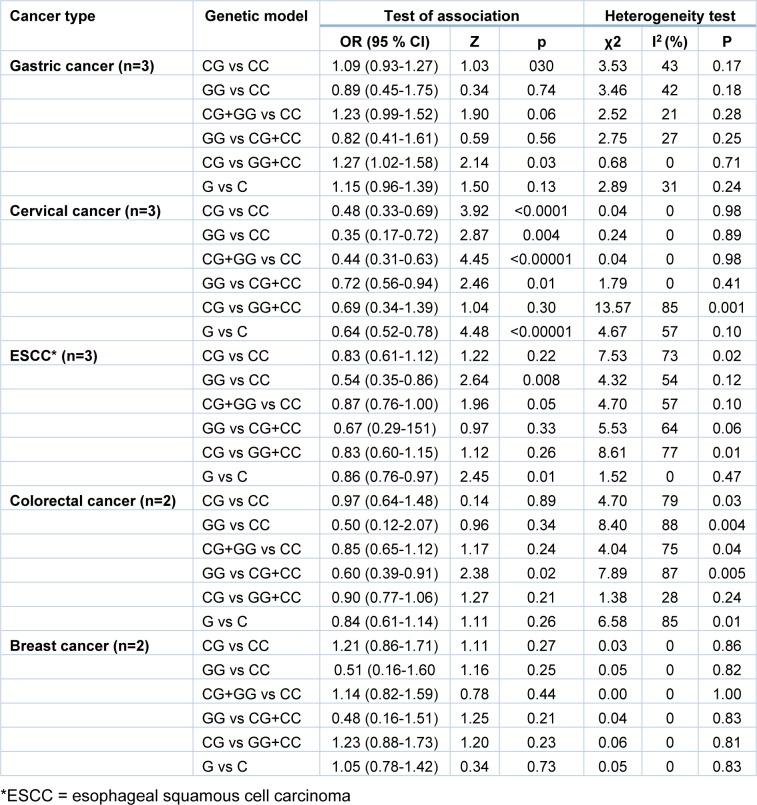
Stratified analysis of miR-124-1 rs531564 variant on susceptibility to cancer
